# Metabolic Pathways for Removing Reactive Aldehydes are Diminished in Atrophic Muscle During Heart Failure

**DOI:** 10.21203/rs.3.rs-3621159/v1

**Published:** 2023-11-21

**Authors:** Mamata Chaudhari, Igor Zelko, Pawel Lorkiewicz, David Hoetker, Benjamin Doelling, Kenneth Brittian, Aruni Bhatnagar, Sanjay Srivastava, Shahid P Baba

**Affiliations:** University of Louisville; University of Louisville; University of Louisville; University of Louisville; University of Louisville; University of Louisville; University of Louisville; University of Louisville; University of Louisville

**Keywords:** Anserine, atrophy, carnosine, heart failure, muscle wasting, ubiquitin proteasome pathway

## Abstract

**Background:**

Muscle wasting is a serious complication in heart failure patients, and oxidative stress is involved in the pathogenesis of muscle wasting. Oxidative stress leads to the formation of toxic lipid peroxidation products, such as 4-hydroxy-2-nonenal (HNE) and acrolein, which causemuscle wasting. In tissues, these toxic aldehydes are metabolically removed by enzymes such asaldo keto reductases and endogenous nucleophiles, such as glutathione and carnosine. Whether these metabolic pathways could be affected in skeletal muscle during heart failure has never been studied.

**Methods:**

Male wild-type C57BL/6J mice were subjected to a pressure overload model of hypertrophy by transaortic constriction (TAC) surgery, and echocardiography was performed after 14 weeks. Different skeletal muscle beds were weighed and analyzed for atrophic and inflammatory markers, *Atrogin1* and *TRIM63, TNF-α* and *IL-6*, respectively, by RT–PCR. Levels of acrolein and HNE-protein adducts, aldehyde-removing enzymes, aldose reductase (AKR1B1) and aldehyde dehydrogenase 2 (ALDH2) were measured by Western blotting, and histidyl dipeptides and histidyl dipeptide aldehyde conjugates were analyzed by LC/MS-MS in the gastrocnemius and soleus muscles of sham- and TAC-operated mice. Furthermore, histidyl dipeptide synthesizing enzyme carnosine synthase (CARNS) and amino acid transporters (PEPT2 and TAUT)wasmeasured in the gastrocnemius muscles of the sham and TAC-operated mice.

**Results:**

TAC-induced heart failure decreases body weight and gastrocnemius and soleus muscle weights. The expression of the atrophic and inflammatory markers *Atrogin1* and TNF-α, respectively, wasincreased (~1.5–2-fold), and the formation of HNE and acrolein-protein adducts was increased in the gastrocnemius muscle of TAC-operated mice. The expression of AKR1B1 remained unchanged, whereas ALDH2 was decreased, in the gastrocnemius muscle of TAC mice. Similarly, in the atrophic gastrocnemius muscle, levels of total histidyl dipeptides (carnosine and anserine) and, in particular,carnosine were decreased. Depletion of histidyl dipeptides diminished the aldehyde removal capacity of the atrophic gastrocnemius muscle. Furthermore, the expression of CARNS and TAUT wasdecreased in the atrophic gastrocnemius muscle.

**Conclusions:**

Collectively, these results show that metabolic pathways involved in the removal of lipid peroxidation products and synthesis of histidyl dipeptides are diminished in atrophic skeletal muscle during heart failure, which could contribute to muscle atrophy.

## Introduction

Heart failure affects up to 1–2% of the total population. Comorbidities such as anemia, chronic kidney disease, chronic obstructive pulmonary disease and diabetes mellitus are extremely common in heart failure patients.^1,2^ Recent evidence shows that skeletal muscle wasting is a serious comorbidity for heart failure patients, both with reduced and preserved ejection fractions.^2^ The prevalence of heart failure-associated muscle wasting is 19–52% among all heart failure patients, which further leads to reduced functional capacity, frequent hospital visits and increased mortality.^1,3,4^ Despite an armamentarium of drugs, such as angiotensin convertase inhibitors or beta-blockers available for heart failure patients, these drugs have a minor benefit in reversing muscle wasting.^5,6^ Although the benefits of testosterone have been documented in improving the exercise capacity of heart failure patients^7–9^, the therapy has not been tested in heart failure patients with cachexia or muscle wasting. Nonetheless, given the strong impact of muscle wasting on mortality in heart failure patients and the lack of effective interventions available to reverse the course of this debilitating syndrome, there is a need for a deeper understanding of the mechanisms that contribute to the pathogenesis of this debilitating syndrome.

The causes of muscle wasting in heart failure are multifactorial and include proinflammatory and immune activation, neurohormonal derangements, poor nutrition, reduced blood flow, oxidative stress, reduced anabolism and increased catabolism. ^1,10–13^ Among these factors, oxidative stress is one of the most common and principal factors that also regulates other mechanisms.^13^ In atrophic muscle, oxidative stress in skeletal muscle activates ubiquitin proteasome system (UPS) proteolytic pathways, the main mechanism involved in protein degradation.^14,15^ Oxidative stress also reduces anabolism in skeletal muscle by oxidizing specific cysteine residues of phosphorylases, such as protein kinase A, which activates AKT and thus decreases protein anabolism.^16^ Oxidative stress induces autophagy, a lysosomal pathway that maintains cell homeostasis by removing damaged cellular components. Under pathological conditions such as fasting, hypoxia and exercise, autophagy increases in association with muscle wasting.^17–19^ In biological tissues, excessive generation of reactive oxygen species leads to the oxidation of membrane lipids, generating several toxic lipid peroxidation products, such as acrolein and 4-hydroxy-2-nonenal (4HNE).^20–22^ These toxic aldehydic products comprise a reactive carbonyl group that reacts with amino acid residues, such as lysine in proteins and nucleophilic sites of DNA, giving rise to a multitude of aldehyde-modified proteins and DNA adducts.^23–25^ The formation of lipid peroxidation products has been widely reported in oxidative stress-associated pathologies, such as atherosclerosis and ischemia–reperfusion.^26–28^ Both lipid peroxidation products and aldehyde-modified proteins trigger pathways that cause muscle wasting, such as inflammation, autophagy, and the UPS.^23,29^ In tissues, lipid peroxidation products are metabolically removed by oxidation and reduction, catalyzed *via* the enzymes aldehyde dehydrogenase (ALDH2) and aldose reductase (AKR1B1), respectively. In addition, reactive aldehydes are also removed by the endogenous nucleophile tripeptide glutathione.^30,31^ and histidyl dipeptides, such as carnosine (b-alanine-histidine).^21,32,33^ Excessive generation of ROS occurs in the atrophic skeletal muscle of heart failure patients,^34^ and the downstream effectors of ROS, namely, lipid peroxidation products, cause muscle wasting. ^35,36^ Therefore, in this study, we investigated whether these toxic lipid peroxidation products are generated and whether their metabolic removal is affected in atrophic skeletal muscle during heart failure. We identified that aldehyde-modified proteins accumulated and that both the enzymatic and nonenzymatic removal of lipid peroxidation products were diminished in atrophic skeletal muscle during heart failure.

## Methods

### Animal housing and maintenance:

Wild-type (WT) C57BL/6J male mice were obtained from Jackson Laboratory (Bar Harbor, Maine) and maintained on normal chow in a pathogen-free facility accredited by the Association for Assessment and Accreditation of Laboratory Animal Care.

### Animal surgeries:

Male C57BL/6J mice were subjected to TAC as described previously.^23^ Briefly, following anesthesia (i.p. 50 mg/kg sodium pentobarbital and 50 mg/kg ketamine hydrochloride), mice were orally intubated and ventilated (oxygen supplement to the room-air inlet) with a mouse ventilator (Hugo Sachs). The aorta was visualized following an intercostal incision. A 7–0 nylon suture was looped around the aorta between the brachiocephalic and left common carotid arteries. The suture was tied around a 27-gauge needle placed adjacent to the aorta to constrict the aorta to a reproducible diameter. The needle was removed, and the chest was closed in layers. Mice were extubated upon recovery of spontaneous breathing. Analgesia (ketoprofen, 5 mg/kg) was provided prior to recovery and by 24 h and 48 h postsurgery. Sham mice were subjected to the same procedure as the TAC cohort except the suture was not tied. Mice in this study were exposed to HEPA- and charcoal-filtered room air (6 h/day, 5 days/week) as mentioned before.^37^

### Echocardiography:

Cardiac function was measured by echocardiography using VisualSonics Vevo 3100 as described previously.^38^ Briefly, mice were anesthetized with 2% isoflurane. The LV end diastolic area, end diastolic average wall thickness and end diastolic volume (EDV), end systolic area (LVESA), end systolic volume (ESV), and ejection fraction (EF) were recorded and calculated.^23^

### Histidyl dipeptides and histidyl dipeptide aldehyde conjugate measurements:

Following 14 weeks of sham and TAC surgeries, all muscle beds, including the gastrocnemius, soleus, tibialis anterior and extensor digitorum longus, were isolated from the sham and TAC-operated mice, weighed and normalized to tibia length. The soleus and gastrocnemius muscles were analyzed for histidyl dipeptides and histidyl dipeptide aldehyde conjugates by UPLC–ESI–MS/MS as described previously.^33,39^

### Protein extraction and immunoblotting:

Gastrocnemius muscles from the sham and TAC mice were homogenized in lysis buffer and centrifuged, and the supernatants were analyzed by Western blots as described previously.^23^ Immunoblots were developed using anti-acrolein (1:1000, LSBio), anti-HNE (1:1000; Abcam), anti-AKR1B1 (1:1000, ABclonal), anti-ALDH2 (1:1000; NOVUSBIO), anti-CARNS (1:1000; COSMOBIO), anti-TAUT (1:1000; ABclonal), and anti-PEPT2 (1:1000; NOVUSBIO) antibodies. Band intensity was quantified by using Image Quant TL software and normalized to Amido-black staining.

### RNA isolation and quantitative real-time PCR:

Total RNA from the gastrocnemius and soleus muscles was isolated by a Qiagen Fibrous tissue RNA mini kit, and the purity of RNA was analyzed using Nanodrop One (Thermo Fisher Scientific) as described previously.^23^ Briefly, cDNA was generated from 2 μg of RNA using Syber Green SuperScript^™^ IV VILO^™^ Master Mix (Thermo Fisher), and PCR was performed using a standard procedure with QuantStudio5 from Applied Biosystems. The expression of the genes encoding *Atrogin1, Murf1, TNF-α, IL-6*, *CARNS, TAUT*, and *PEPT2* was determined using quantitative RT–PCR. The results were normalized to the 18S ribosome and expressed according to the comparative Ct method, where the Ct values of the gene of interest were compared to the controls.

### Statistical analysis.

Data are presented as the mean±SEM. Sham and TAC groups were analyzed using one-way analysis of variance followed by Bonferroni correction or Student’s *t* test. Statistical significance was accepted at p<0.05.

## Results

### Transverse aortic constriction (TAC)-induced cardiac dysfunction

**Figure 1:**
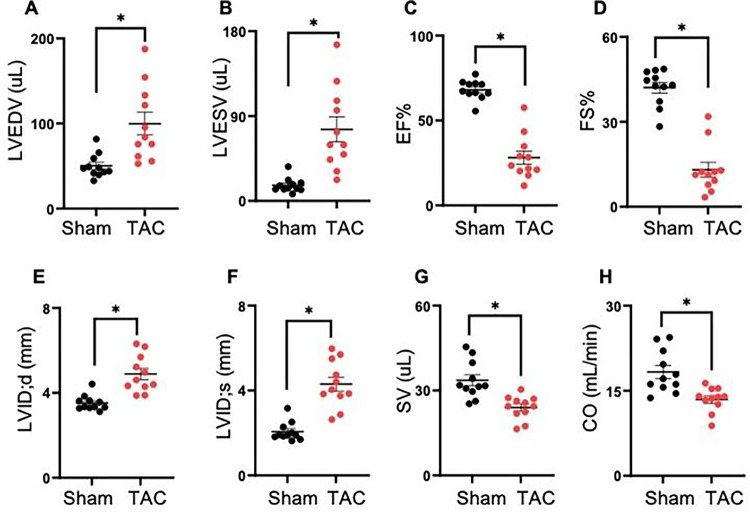
Transverse aortic constriction (TAC) induces cardiac dysfunction Wild-type C57BL/6J mice were subjected to sham and TAC surgeries for 14 weeks. **(A)** End-diastolic volume (EDV), **(B)**end-systolic volume (ESV), **(C)** ejection fraction (EF), **(D)**fractional shortening (FS), **(E)** left ventricular internal diameter in diastole (LVIDd), **(F)** left ventricular internal diameter in systole (LVIDs), **(G)** stroke volume (SV) and **(H)** cardiac output (CO). Data are presented as the mean ± SEM, n=10–11 mice in each group, *p<0.001 vs sham.

Mice were subjected to sham and TAC surgeries. TAC-operated mice developed significant left ventricular dilation, as indicated by the significant increase in end-diastolic volume (sham; EDV: 50 ± 13.0 vs TAC 99 ± 43, p = 0.001), end-systolic volume (sham; ESV: 16 ± 7.6 vs TAC 75 ± 43 μL, p < 0.002), and decrease in ejection fraction (sham; EF: 68 ± 5.7 vs TAC 28 ± 13.0, p < 0.001) and fractional shortening (sham; FS: 42 ± 6.3 vs TAC 13 ± 8.5%, p = 0.001) compared with sham mice (Fig. 1A–D). Furthermore, in TAC mice, the left ventricular internal diameter in diastole (sham; LVIDd: 3.5 ± 0.3 vs TAC 4.8 ± 0.8 mm, p = 0.0001), left ventricular internal diameter in systole (sham; LVIDs: 2.0 ± 0.4 vs TAC 4.3 ± 1.0 mm, p < 0.003) were increased, and the stroke volume (sham; SV: 33.6 ± 6.5 vs TAC 23.9 ± 4.2 μL, p < 0.005) and cardiac output (sham; CO: 18.3 ± 3.7 vs TAC 13.4 ± 2.1 mL/min, p < 0.001) were decreased compared with sham mice (Fig. 1E–H).

### Transverse aortic constriction (TAC) of the mouse heart causes muscle wasting

**Figure 2:**
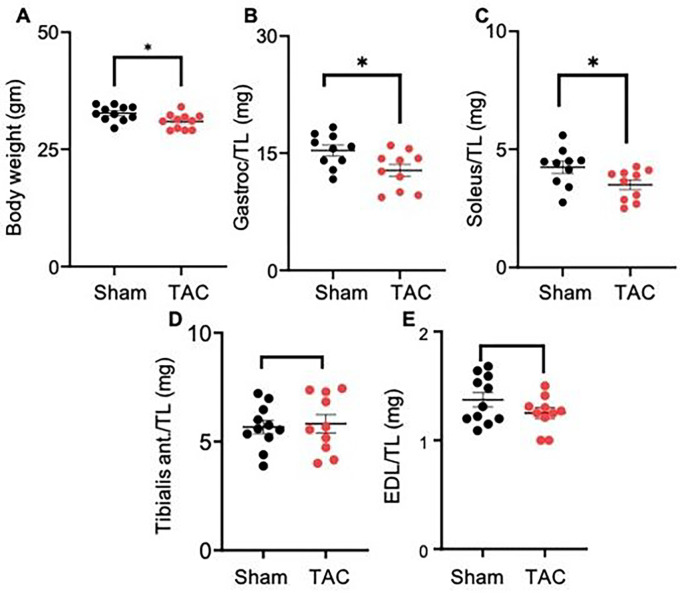
Transaortic constriction (TAC)-induced heart failure decreases body weight and muscle mass. Wild-type C57BL/6J mice were subjected to sham and TAC surgeries for 14 weeks. (**A**) Body weight, (**B**) gastrocnemius (gastroc.) muscle weight, (**C**) soleus muscle weight, (**D**) tibialis anterior muscle weight, (**E**) extensor digitorum longus (EDL) muscle weight. Muscle weights were normalized to tibia length. Data are shown as the mean ± SEM, n=10 mice in each group,*p<0.02 vs sham-operated mice.

**Figure 3:**
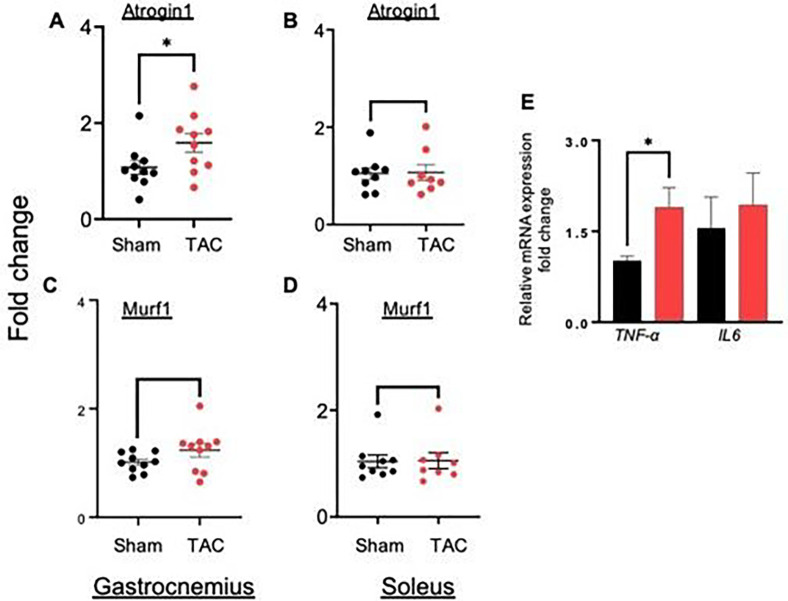
Atrophic and inflammatory gene expression isincreased in the gastrocnemius muscle of heart failure mice. Wild-type C57BL/6J mice were subjected to sham and transaortic constriction (TAC) for 14 weeks. Fold change in the expression of atrophic genes, *atrogin1* and *Murf1* in (**A, C**) gastrocnemius and (**B, D**) soleus muscles. (**C**) Inflammatory genes *TNF-α* and *IL-6*in the gastrocnemius muscle. Data are shown as the mean ± SEM, *p<0.05, n=9–10 in each group.

In the TAC-operated mice, body weight (sham: 32.76 ± 1.6 vs TAC 30.96 ± 1.6 gm, p < 0.019), gastrocnemius muscle weight (sham: 15.34 ± 2.0 vs TAC: 12.79 ± 2.0 mg, p < 0.02) and soleus muscle weight (sham: 4.24 ± 0.80 vs TAC 3.50 ± 0.62 mg, p = 0.04) were significantly decreased compared with those of the sham-operated mice (Fig. 2A–C). The weights of the tibialis anterior (sham: 5.75 ± 1.04 vs TAC: 5.82 ± 1.20 mg) and extensor digitorum longus (sham: 1.39 ± 0.22 vs TAC: 1.25 ± 0.15 mg) remained unchanged between the sham and TAC-operated mice (Fig. 2D–E). Because the gastrocnemius and soleus muscle weights were decreased, we next measured the expression of atrophy-related genes in these muscle beds only. In the gastrocnemius muscle of TAC-operated mice, *Atrogin1* expression increased ~1.5-fold (p < 0.05) compared with that in the sham-operated mice, whereas the expression of another atrophic marker, *Trim63/MURF1*, was increased but unable to reach statistical significance (Fig. 3A–D). Although soleus muscle weight was decreased, the expression of atrophic markers remained unchanged between the sham and TAC-operated mice.

### Heart failure increases inflammation in the gastrocnemius muscle

To examine whether heart failure triggers the inflammatory pathway in the gastrocnemius muscle, we measured the expression of the inflammation-related genes tumor necrosis factor alpha (*TNF-α*) and interleukin 6 (*IL-6*) and found that *TNF-α* expression was increased ~ 1–2-fold (p = 0.004) compared with that in sham-operated mice, whereas the expression of *IL-6* remained unchanged (Fig. 3E).

### Heart failure increases carbonyl stress in the gastrocnemius muscle

**Figure 4:**
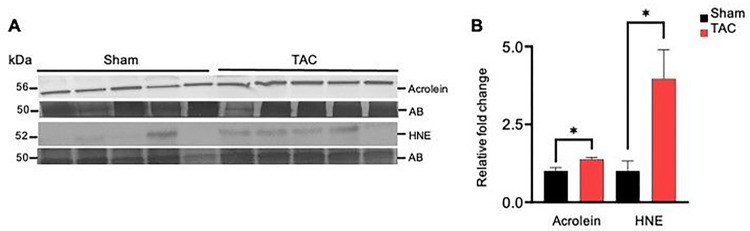
Heart failure increases the accumulation of aldehyde protein adducts in the gastrocnemius muscle. Gastrocnemius muscles from wild-type C57BL/6J mice subjected to sham and transaortic constriction (TAC) were analyzed by Western blotting for aldehyde-modified protein adducts. Representative blots for (**A**) acrolein and HNE protein adducts and amido black (AB).(**B**) Bar graph shows the intensity of bands normalized to amido black, *p<0.05 vs sham. Data are shown as the mean ± SEM, *n* = 6 samples in each group.

To examine whether heart failure affects the generation of lipid peroxidation products in the muscle, we performed Western blot analysis of the gastrocnemius muscle using anti-acrolein and anti-HNE antibodies. Formation of both the acrolein and HNE protein adducts was increased ~2–3-fold in the gastrocnemius muscle of the TAC compared with the sham-operated mice (p < 0.05; Fig. 4A–B). Collectively, these results suggest that uncontrolled generation of reactive oxygen species in skeletal muscle during heart failure increases the formation of aldehyde protein adducts in the atrophic gastrocnemius muscle.

### Aldehyde removal pathways are diminished in the atrophic gastrocnemius muscle

**Figure 5:**
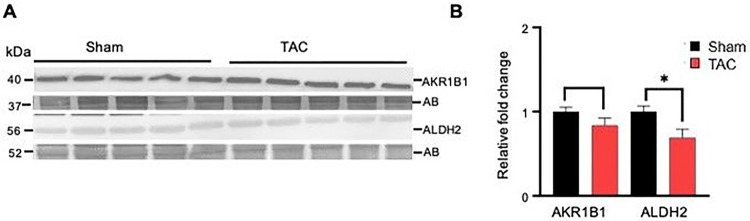
Heart failure decreases the expression of aldehyde dehydrogenase (ALDH2) in the gastrocnemius muscle. Gastrocnemius muscles from wild-type C57BL/6J mice subjected to sham and transaortic constriction were analyzed by Western blotting. Representative blots for **(A)** aldehyde dehydrogenase (ALDH2) and aldose reductase (AKR1B1), normalized to amido black (AB). **(B)** Bar graphs show the intensity of bands normalized to amido black (AB), *p<0.02 vs sham. Data are shown as the mean ± SEM, n=6 samples in each group.

To determine whether the processes that remove lipid peroxidation products are affected in the gastrocnemius muscle during heart failure, we first compared the expression of enzymes aldose reductase (AKR1B1) and aldehyde dehydrogenase (ALDH2) between the sham and TAC-operated mice. The expression of AKR1B1 remained unchanged, whereas ALDH2 was decreased (p < 0.02) in the TAC mice compared with the sham mice (Fig. 5A–B).

**Figure 6:**
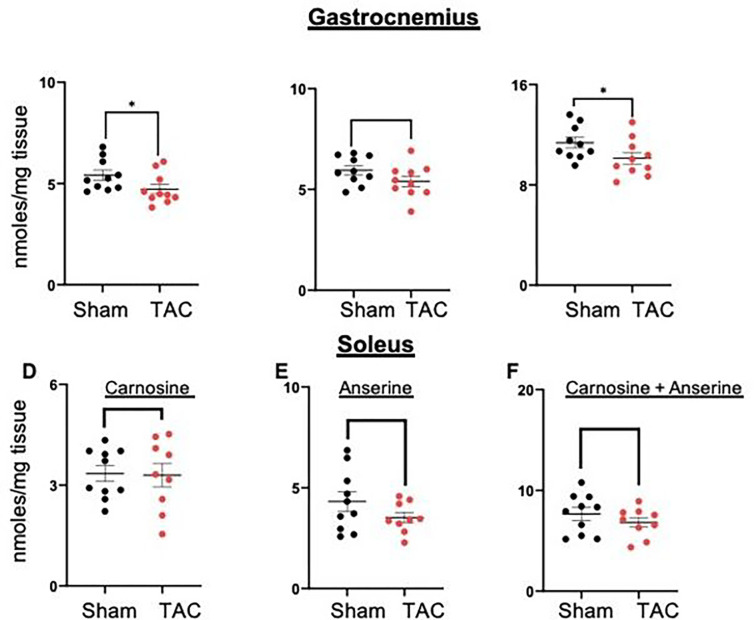
Histidyl dipeptide levels are depleted in the atrophic gastrocnemius muscle. Gastrocnemius and soleus muscles collected from the sham (*n* = 10) and TAC mice (*n* = 11) were analyzed by LC–MS/MS for different histidyl dipeptides. Levels of carnosine in (**A**) gastrocnemius and (**D**) soleus muscles, anserine in (**B**) gastrocnemius and (**E**) soleus muscles, and total histidyl dipeptides in (**C**) gastrocnemius and (**F**) soleus muscles. Data are shown as the mean ± SEM and *p<0.02 vs sham.

Next, to examine whether heart failure affects histidyl dipeptides in the gastrocnemius muscle, we measured carnosine and anserine levels by LC/MS-MS. The levels of carnosine were significantly decreased in the gastrocnemius muscle of TAC mice compared with sham mice (sham: 5.76 ± 1.3 vs TAC: 4.72 ± 0.75 nmoles/mg tissue, p < 0.04, Fig. 6A). Anserine levels were also decreased but unable to reach statistical significance (sham: 6.20 ± 1.08 vs TAC: 5.42 ± 0.82 nmoles/mg tissue, p = 0.07, Fig. 6B). Total histidyl dipeptides (carnosine and anserine) were significantly lower in the gastrocnemius muscle of TAC-operated mice (sham: 11.97 ± 1.5 vs TAC: 10.13 ± 1.4 nmoles/mg tissue, p < 0.05, Fig. 6C). In the soleus muscle, carnosine and anserine levels remained unchanged between the sham and TAC mice (Fig. 6D–F).

**Figure 7:**
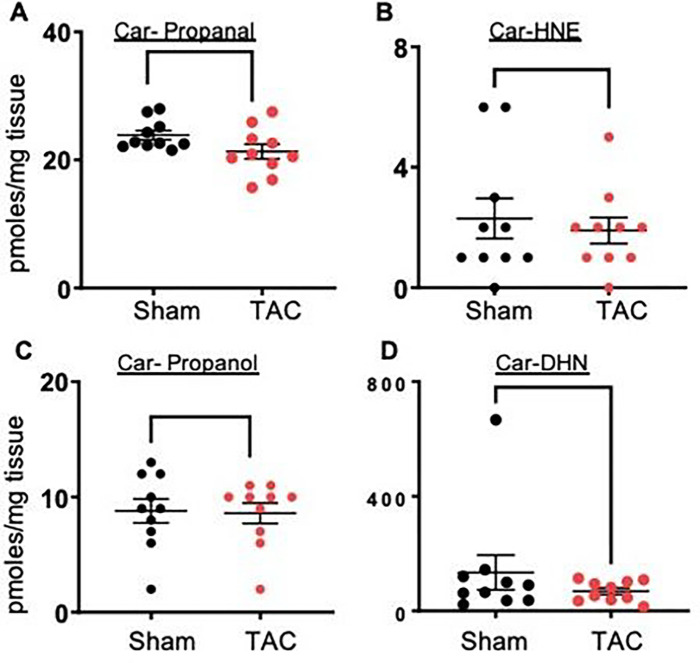
Carnosine aldehyde conjugate formation in the gastrocnemius muscle. Levels of (**A**) carnosine propanal, (**B**) carnosine HNE, (**C**) carnosine propanol and (**D**) carnosine DHN in the gastrocnemius muscle of the sham and TAC-operated C57BL/6J mice after 14 weeks. Data are shown as the mean ± SEM, n=10–11 in each group, *p<0.07 vs sham.

Next, we examined whether the depletion of histidyl dipeptides in skeletal muscle could affect the removal of lipid peroxidation products and measured carnosine aldehyde conjugates, carnosine-propanal, carnosine-propanol, carnosine-HNE and carnosine-DHN, by LC–MS/MS. Levels of carnosine-propanal in the gastrocnemius muscle of TAC mice tended to decrease (sham: 24.12 ± 2.3 vs TAC 21.30 ± 3.0 pmoles/mg tissue, p = 0.07). Carnosine-propanal, carnosine-HNE and carnosine-DHN remained unchanged between the sham and TAC mice. Collectively, both the enzymatic and nonenzymatic pathways that remove reactive aldehydes are diminished in the atrophic gastrocnemius muscle during heart failure (Fig. 7A–D).

### Histidyl dipeptide synthesis and transport were decreased in the gastrocnemius muscle during heart failure

**Figure 8:**
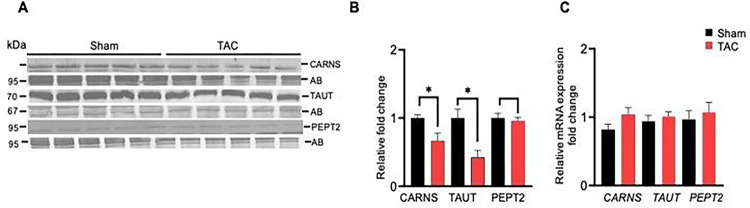
The expression of carnosine synthase and TAUT is decreased in the gastrocnemius muscle of heart failure mice. Representative Western blots developed from the homogenates of gastrocnemius muscles of sham and trans aortic constriction (TAC)-operated mice. (**A**) Blots were developed using anticranosine synthase (CARNS), antihuman synthetic taurine transporter (TAUT), and antihuman peptide transporter (PEPT2) antibodies. (**B**) Relative mRNA expression of CARNS, TAUT and *PEPT2* in the gastrocnemius muscle. (**C**) Data are shown as the mean ± SEM, n=5–6 in each group, *p<0.04 vs sham.

Finally, we examined how histidyl dipeptide levels in the gastrocnemius muscle are decreased during heart failure and compared the expression of carnosine synthesizing enzyme, carnosine synthase (CARNS), and amino acid transporters (TAUT and PEPT2) between sham and TAC mice. The expression of CARNS and TAUT were decreased, whereas PEPT2 remained unchanged in the gastrocnemius muscle of TAC mice (Fig. 8A–B). To further examine whether the decrease in CARNS and TAUT occurs at the mRNA level, we performed RT–PCR and found that *CARNS, PEPT2* and *TAUT* expression remained unchanged between the sham and TAC mice (Fig. 8). C). Taken together, these results suggest that the decrease in histidyl dipeptide synthesis and transport of amino acids could contribute to diminishing the gastrocnemius muscle histidyl dipeptides during heart failure.

## Discussion

In this study, we report that the gastrocnemius and soleus muscles undergo atrophy during heart failure. Acrolein and HNE-protein adducts accumulate, and the expression of the aldehyde-removing enzyme aldehyde dehydrogenase (ALDH2) is decreased in the atrophic gastrocnemius muscle. Levels of endogenous histidyl dipeptides, especially carnosine, which conjugates with different reactive aldehydes, were decreased in the gastrocnemius muscle of heart failure mice. Protein expression of the enzyme CARNS, which synthesizes carnosine, and the amino acid transporter TAUT were decreased in the gastrocnemius muscle during heart failure, suggesting that decreases in both the synthesis and the transport of essential amino acids needed for histidyl dipeptide synthesis could contribute to histidyl dipeptide depletion in the atrophic muscle. The distinct decrease in histidyl dipeptide synthesis and ALDH2 in the gastrocnemius muscle suggests that derangements in these aldehyde-removing pathways might be specifically involved in the increased formation of aldehyde protein adducts in the gastrocnemius muscle, which could trigger inflammation and muscle wasting during heart failure.

Muscle wasting is a serious complication affecting a sizable proportion of heart failure patients. In heart failure patients, the prevalence of muscle wasting is ~20% higher than that in age-matched normal humans.^4^ Recent reports show that muscle wasting prevalence is also higher in younger heart failure patients.^40^ Muscle wasting and impaired skeletal muscle function following heart failure play a key role in the development of exercise intolerance, fatigue, a decrease in the distance covered on a six-minute walk test and hand grip strength.^3,4,41,42^ In this study, we found that mice subjected to TAC-induced heart failure had decreased body weight and gastrocnemius and soleus muscle weights. Gene expression of the muscle-specific ubiquitin ligase *Atrogin1*, a marker of atrophy, was increased in the gastrocnemius muscle only, indicating that among the different muscle beds, the gastrocnemius muscle undergoes atrophy in this model of heart failure. Our results are in contrast to the previous work by Szaroszyk et al. ^43^ showing that TAC-induced heart failure for 12–14 weeks reduced the weight of all muscle beds, including the quadriceps, gastrocnemius, triceps, and soleus. This discrepancy could be due to the handling of the mice. In our study, both the sham and TAC-operated mice were exposed to HEPA- and charcoal-filtered room air.^37^ Nonetheless, TAC decreased body weight and induced muscle wasting, suggesting that the TAC model of heart failure in mice replicates the muscle wasting syndrome of heart failure patients.

The mechanisms by which heart failure induces muscle wasting are not clear, and currently, no therapies are available that can stop the progression of muscle wasting in heart failure patients. One of the common features associated with muscle wasting is the release of atrophic factors from the diseased tissue, such as angiotensin II. Patients with heart failure have increased levels of circulating angiotensin and decreased blood flow to skeletal muscle, which induces oxidative stress.^44–46^ Increased levels of oxidative stress markers have been documented in the skeletal muscle of chronic heart failure patients, which correlates with reduced exercise capacity and lower antioxidant levels.^34^ While the formation of reactive oxygen species (ROS) is tightly controlled in biological systems, the deregulation of redox homeostasis has emerged as a common pathogenic mechanism in age- and cancer-related muscle loss.^47^ When ROS formation increases, antioxidant defenses become overwhelmed, resulting in the induction of a wide variety of lipid peroxidation products, such as acrolein and 4-hydroxy-trans-2-nonenal (4-HNE), which can covalently bind with proteins and DNA.^23^ In this study, we found that the acrolein and HNE-protein adducts were increased in the gastrocnemius muscle of TAC-operated mice, indicating that heart failure overwhelms both the redox and aldehyde removal homeostasis in skeletal muscle, thus resulting in the accumulation of 4-HNE- and acrolein-modified proteins. Previous works have shown that acrolein induces myotube atrophy and inhibits myogenic differentiation in myoblasts.^36^ Acrolein exposure also decreases muscle weight and retards muscle regeneration in mice.^48^ Similarly, increased formation of HNE protein adducts occurs in the gastrocnemius muscle of mice with the progression of age^35,^ and preventing the accumulation of HNE in the gastrocnemius muscle alleviates muscle atrophy.^49^ In this context, accumulation of the acrolein and HNE protein adducts in skeletal muscle could contribute to aggravating muscle wasting syndrome during heart failure. Previous reports have shown that aldehyde-modified proteins behave as damage-associated molecular patterns (DAMPs) that alarm the immune system by inducing adaptive immune responses.^50^ In particular, different human pathologies associated with oxidative stress, such as atherosclerosis, show that aldehyde-modified proteins activate adaptive immune responses.^51–53^ Our results show that TNF-α expression was increased in atrophic skeletal muscle, suggesting that the formation of aldehyde-modified DAMPs might activate inflammation in atrophic skeletal muscle under heart failure conditions. Therefore, future studies are needed to determine the contribution of these aldehyde-modified DAMPs generated in the gastrocnemius muscle to immune modulating activities and muscle atrophy under heart failure conditions.

Recent reports have shown that a missense single nucleotide polymorphism in the aldehyde dehydrogenase 2 (ALDH2) gene, rs671 (ALDH2*2), increases 4-HNE formation in skeletal muscle and promotes muscle atrophy. ^54^ ALDH2 deficiency also promotes age-related muscle atrophy, increases the formation of HNE adducts^55^ and treatment with antioxidants such as Vit. E and Chlorella rescues the genetic and age-induced risk of atrophy.^49,54^ On the other hand, overexpression of ALDH2 in skeletal muscle reverses oxidative stress and muscle atrophy due to exhaustive exercise.^56^ We investigated whether the accumulation of aldehyde-modified proteins in the gastrocnemius during heart failure is also associated with the derangements of mechanisms that remove reactive aldehydes. Our results showed that ALDH2 expression was decreased, and the formation of aldehyde-modified protein adducts was increased in the gastrocnemius muscle of heart failure mice. Thus, the decrease in the expression of ALDH2 could contribute to the accumulation of aldehyde-modified proteins and consequently lead to muscle wasting. Extensive evidence shows that activation of ALDH2 by a small molecular weight activator of ALDH2 prevents the accumulation of aldehydes in ischemic tissues and exerts protective action against acute ischemic injury in the heart and brain.^57–59^ Therefore, activation of ALDH2 by a selective ALDH2 activator may offer benefits by removing toxic aldehydes from skeletal muscle and exerting protection from heart failure-induced muscle wasting.

In skeletal muscle, especially the gastrocnemius muscle, there are high levels of histidyl dipeptides, such as carnosine and anserine.^39^ Among these histidyl dipeptides, carnosine is present in humans, whereas anserine is found in rodents.^33,39^ These dipeptides are synthesized by the enzymes carnosine synthase (CARNS) and carnosine methyltransferase.^33,60–62^ Histidyl dipeptides exhibit a unique chemistry, where the amino group of the b-alanine can bind with reactive aldehydes *via* Michael adducts or Schiff’s base. They also exhibit the ability to quench reactive oxygen species, buffer intracellular pH and chelate first transition metals.^63,64^ Among all the nucleophiles present in skeletal muscle, only histidyl dipeptide levels can be increased either by exercise or by supplementing the precursor amino acid b-alanine.^39^ Because of their multifunctionality and the ease with which these dipeptides can be increased in different tissues, supplementation with b-alanine is widely used to improve exercise capacity.^39,65^ Previously, we showed that increasing carnosine levels in the skeletal muscle of humans by β-alanine supplementation enhances the removal of reactive aldehydes from skeletal muscle.^39^ Furthermore, we also found that carnosine levels were decreased in the skeletal muscle of cancer cachexia patients.^66^ Recent reports have shown that carnosine supplementation improves the exercise capacity of heart failure patients and glucose homeostasis in type 2 diabetics.^67,68^ Therefore, given the multitude of benefits of carnosine associated with maintaining skeletal muscle function and our observations showing that carnosine and total histidyl dipeptides were depleted in the gastrocnemius muscle of heart failure mice, these dipeptides are essential for skeletal muscle health during heart failure.

Histidyl dipeptide homeostasis within skeletal muscle is maintained by a complex array of transporters, such as TAUT and PEPT, which are synthesized by CARNS and bind with lipid peroxidation products.^66,69^ We found that the expression of the enzyme CARNS and transporter TAUT was decreased in the gastrocnemius muscle of heart failure mice, suggesting that both the synthesis and transport of amino acids needed for carnosine synthesis are affected and could contribute to histidyl dipeptide depletion during heart failure. Interestingly, our results show that decreases in CARNS and TAUT protein expression were not mimicked at the mRNA level, suggesting that CARNS and TAUT might be targets of the protein degradation machinery activated during muscle wasting. Nonetheless, how CARNS and TAUT protein expression are decreased needs to be studied. Paralleling the decrease in carnosine synthesis was the trending diminished removal of reactive aldehydes in the gastrocnemius muscle of heart failure mice. Overall, both the enzymatic and nonenzymatic aldehyde removal pathways become defective in skeletal muscle during heart failure and thus could contribute to triggering muscle wasting.

## Conclusion

In conclusion, our results support that a murine model of TAC-induced heart failure causes muscle wasting. Lipid peroxidation products, the downstream toxic products of oxidative stress, accumulate and result in the formation of acrolein and HNE-modified protein adducts in the atrophic gastrocnemius muscle. In addition, the metabolic processes of aldehyde removal are defective in the atrophic gastrocnemius muscle. Therefore, the main observation of our study showing that endogenous histidyl dipeptides are depleted in the atrophic gastrocnemius muscle and that these dipeptides can quench ROS, form conjugates with reactive aldehydes and can be replenished in the muscle by supplementation^21,39^ opens an opportunity to test how these dipeptides could contribute to muscle wasting and whether maintaining or increasing their synthesis in skeletal muscle could prevent or reverse muscle wasting syndrome during heart failure.

## Data Availability

All generated and analyzed data for this manuscript are included in this article.

## Abbreviations

TAC: Transverse aortic constriction
ROS: reactive oxygen species
AKR1B1: aldose reductase
ALDH2: aldehyde dehydrogenase
CARNS: carnosine synthase
PEPT2: human peptide transporter
TAUT: taurine transporter
TNF-α: tumor necrosis factor alpha
IL-6: interleukin 6

## References

[1] von Haehling S, Ebner N, Dos Santos MR, Springer J, Anker SD. Muscle wasting and cachexia in heart failure: mechanisms and therapies. Nat Rev Cardiol. 2017;14:323–341. doi: 10.1038/nrcardio.2017.51 28436486

[2] Ponikowski P, Voors AA, Anker SD, Bueno H, Cleland JG, Coats AJ, Falk V, Gonzalez-Juanatey JR, Harjola VP, Jankowska EA, 2016 ESC Guidelines for the diagnosis and treatment of acute and chronic heart failure: The Task Force for the diagnosis and treatment of acute and chronic heart failure of the European Society of Cardiology (ESC). Developed with the special contribution of the Heart Failure Association (HFA) of the ESC. Eur J Heart Fail. 2016;18:891–975. doi: 10.1002/ejhf.592 27207191

[3] Bekfani T, Pellicori P, Morris DA, Ebner N, Valentova M, Steinbeck L, Wachter R, Elsner S, Sliziuk V, Schefold JC, Sarcopenia in patients with heart failure with preserved ejection fraction: Impact on muscle strength, exercise capacity and quality of life. Int J Cardiol. 2016;222:41–46. doi: 10.1016/j.ijcard.2016.07.135 27454614

[4] Fulster S, Tacke M, Sandek A, Ebner N, Tschope C, Doehner W, Anker SD, von Haehling S. Muscle wasting in patients with chronic heart failure: results from the studies investigating comorbidities aggravating heart failure (SICA-HF). Eur Heart J. 2013;34:512–519. doi: 10.1093/eurheartj/ehs381 23178647

[5] Hryniewicz K, Androne AS, Hudaihed A, Katz SD. Partial reversal of cachexia by beta-adrenergic receptor blocker therapy in patients with chronic heart failure. J Card Fail. 2003;9:464–468. doi: 10.1016/s1071-9164(03)00582-7 14966787

[6] Lainscak M, Keber I, Anker SD. Body composition changes in patients with systolic heart failure treated with beta blockers: a pilot study. Int J Cardiol. 2006;106:319–322. doi: 10.1016/j.ijcard.2005.01.061 16337039

[7] Pugh PJ, Jones TH, Channer KS. Acute hemodynamic effects of testosterone in men with chronic heart failure. Eur Heart J. 2003;24:909–915. doi: 10.1016/s0195-668x(03)00083-6 12714022

[8] Malkin CJ, Pugh PJ, West JN, van Beek EJ, Jones TH, Channer KS. Testosterone therapy in men with moderate severity heart failure: a double-blind randomized placebo controlled trial. Eur Heart J. 2006;27:57–64. doi: 10.1093/eurheartj/ehi443 16093267

[9] Caminiti G, Volterrani M, Iellamo F, Marazzi G, Massaro R, Miceli M, Mammi C, Piepoli M, Fini M, Rosano GM. Effect of long-acting testosterone treatment on functional exercise capacity, skeletal muscle performance, insulin resistance, and baroreflex sensitivity in elderly patients with chronic heart failure a double-blind, placebo-controlled, randomized study. J Am Coll Cardiol. 2009;54:919–927. doi: 10.1016/j.jacc.2009.04.078 19712802

[10] Okutsu M, Call JA, Lira VA, Zhang M, Donet JA, French BA, Martin KS, Peirce-Cottler SM, Rembold CM, Annex BH, Yan Z. Extracellular superoxide dismutase ameliorates skeletal muscle abnormalities, cachexia, and exercise intolerance in mice with congestive heart failure. Circ Heart Fail. 2014;7:519–530. doi: 10.1161/CIRCHEARTFAILURE.113.000841 24523418 PMC4080303

[11] Egerman MA, Glass DJ. Signaling pathways controlling skeletal muscle mass. Crit Rev Biochem Mol Biol. 2014;49:59–68. doi: 10.3109/10409238.2013.857291 24237131 PMC3913083

[12] Glass DJ. Signaling pathways perturbing muscle mass. Curr Opin Clin Nutr Metab Care. 2010;13:225–229. doi: 10.1097/mco.0b013e32833862df 20397318

[13] Abrigo J, Elorza AA, Riedel CA, Vilos C, Simon F, Cabrera D, Estrada L, Cabello-Verrugio C. Role of Oxidative Stress as Key Regulator of Muscle Wasting during Cachexia. Oxid Med Cell Longev. 2018;2018:2063179. doi: 10.1155/2018/2063179 29785242 PMC5896211

[14] Sandri M. Protein breakdown in muscle wasting: role of autophagy–lysosome and ubiquitin–proteasome. Int J Biochem Cell Biol. 2013;45:2121–2129. doi: 10.1016/j.biocel.2013.04.023 23665154 PMC3775123

[15] Bilodeau PA, Coyne ES, Wing SS. The ubiquitin proteasome system in atrophying skeletal muscle: roles and regulation. Am J Physiol Cell Physiol. 2016;311:C392–403. doi: 10.1152/ajpcell.00125.2016 27510905

[16] Cross JV, Templeton DJ. Regulation of signal transduction through protein cysteine oxidation. Antioxid Redox Signal. 2006;8:1819–1827. doi: 10.1089/ars.2006.8.1819 16987034

[17] Dobrowolny G, Aucello M, Rizzuto E, Beccafico S, Mammucari C, Boncompagni S, Belia S, Wannenes F, Nicoletti C, Del Prete Z, Skeletal muscle is a primary target of SOD1G93A-mediated toxicity. Cell Metab. 2008;8:425–436. doi: 10.1016/j.cmet.2008.09.002 19046573

[18] Rahman M, Mofarrahi M, Kristof AS, Nkengfac B, Harel S, Hussain SN. Reactive oxygen species regulation of autophagy in skeletal muscles. Antioxid Redox Signal. 2014;20:443–459. doi: 10.1089/ars.2013.5410 24180497

[19] Rodney GG, Pal R, Abo-Zahrah R. Redox regulation of autophagy in skeletal muscle. Free Radic Biol Med. 2016;98:103–112. doi: 10.1016/j.freeradbiomed.2016.05.010 27184957 PMC4975974

[20] Baba SP, Hellmann J, Srivastava S, Bhatnagar A. Aldose reductase (AKR1B3) regulates the accumulation of advanced glycosylation end products (AGEs) and the expression of AGE receptor (RAGE). Chem Biol Interact. 2011;191:357–363. doi: 10.1016/j.cbi.2011.01.024 21276777 PMC3145413

[21] Baba SP, Hoetker JD, Merchant M, Klein JB, Cai J, Barski OA, Conklin DJ, Bhatnagar A. Role of aldose reductase in the metabolism and detoxification of carnosine-acrolein conjugates. J Biol Chem. 2013;288:28163–28179. doi: 10.1074/jbc.M113.504753 23928303 PMC3784727

[22] Conklin DJ, Guo Y, Jagatheesan G, Kilfoil PJ, Haberzettl P, Hill BG, Baba SP, Guo L, Wetzelberger K, Obal D, Genetic Deficiency of Glutathione S-Transferase P Increases Myocardial Sensitivity to Ischemia–Reperfusion Injury. Circ Res. 2015;117:437–449. doi: 10.1161/CIRCRESAHA.114.305518 26169370 PMC4854443

[23] Baba SP, Zhang D, Singh M, Dassanayaka S, Xie Z, Jagatheesan G, Zhao J, Schmidtke VK, Brittian KR, Merchant ML, Deficiency of aldose reductase exacerbates early pressure overload-induced cardiac dysfunction and autophagy in mice. J Mol Cell Cardiol. 2018;118:183–192. doi: 10.1016/j.yjmcc.2018.04.002 29627295 PMC6205513

[24] Barrera G, Pizzimenti S, Ciamporcero ES, Daga M, Ullio C, Arcaro A, Cetrangolo GP, Ferretti C, Dianzani C, Lepore A, Gentile F. Role of 4-hydroxynonenal-protein adducts in human diseases. Antioxid Redox Signal. 2015;22:1681–1702. doi: 10.1089/ars.2014.6166 25365742

[25] Liu X, Lovell MA, Lynn BC. Detection and quantification of endogenous cyclic DNA adducts derived from trans-4-hydroxy-2-nonenal in human brain tissue by isotope dilution capillary liquid chromatography nanoelectrospray tandem mass spectrometry. Chem Res Toxicol. 2006;19:710–718. doi: 10.1021/tx0502903 16696574

[26] Barski OA, Xie Z, Baba SP, Sithu SD, Agarwal A, Cai J, Bhatnagar A, Srivastava S. Dietary carnosine prevents early atherosclerotic lesion formation in apolipoprotein E-null mice. Arterioscler Thromb Vasc Biol. 2013;33:1162–1170. doi: 10.1161/ATVBAHA.112.300572 23559625 PMC3869200

[27] Srivastava S, Vladykovskaya E, Barski OA, Spite M, Kaiserova K, Petrash JM, Chung SS, Hunt G, Dawn B, Bhatnagar A. Aldose reductase protects against early atherosclerotic lesion formation in apolipoprotein E-null mice. Circ Res. 2009;105:793–802. doi: 10.1161/CIRCRESAHA.109.200568 19729598 PMC3548455

[28] Kaiserova K, Srivastava S, Hoetker JD, Awe SO, Tang XL, Cai J, Bhatnagar A. Redox activation of aldose reductase in the ischemic heart. J Biol Chem. 2006;281:15110–15120. doi: 10.1074/jbc.M600837200 16567803

[29] Hill BG, Haberzettl P, Ahmed Y, Srivastava S, Bhatnagar A. Unsaturated lipid peroxidation-derived aldehydes activate autophagy in vascular smooth-muscle cells. Biochem J. 2008;410:525–534. doi: 10.1042/BJ20071063 18052926

[30] Srivastava S, Dixit BL, Cai J, Sharma S, Hurst HE, Bhatnagar A, Srivastava SK. Metabolism of lipid peroxidation product, 4-hydroxynonenal (HNE) in rat erythrocytes: role of aldose reductase. Free Radic Biol Med. 2000;29:642–651. 11033416 10.1016/s0891-5849(00)00351-8

[31] Srivastava S, Watowich SJ, Petrash JM, Srivastava SK, Bhatnagar A. Structural and kinetic determinants of aldehyde reduction by aldose reductase. Biochemistry. 1999;38:42–54. doi: 10.1021/bi981794l 9890881

[32] Blancquaert L, Baba SP, Kwiatkowski S, Stautemas J, Stegen S, Barbaresi S, Chung W, Boakye AA, Hoetker JD, Bhatnagar A, Carnosine and anserine homeostasis in skeletal muscle and heart is controlled by beta-alanine transamination. J Physiol. 2016;594:4849–4863. doi: 10.1113/JP272050 27062388 PMC5009790

[33] Zhao J, Conklin DJ, Guo Y, Zhang X, Obal D, Guo L, Jagatheesan G, Katragadda K, He L, Yin X, Cardiospecific Overexpression of ATPGD1 (Carnosine Synthase) Increases Histidine Dipeptide Levels and Prevents Myocardial Ischemia–Reperfusion Injury. J Am Heart Assoc. 2020;9:e015222. doi: 10.1161/JAHA.119.015222 32515247 PMC7429021

[34] Linke A, Adams V, Schulze PC, Erbs S, Gielen S, Fiehn E, Mobius-Winkler S, Schubert A, Schuler G, Hambrecht R. Antioxidative effects of exercise training in patients with chronic heart failure: increase in radical scavenger enzyme activity in skeletal muscle. Circulation. 2005;111:1763–1770. doi: 10.1161/01.CIR.0000165503.08661.E5 15809365

[35] Braga M, Sinha Hikim AP, Datta S, Ferrini MG, Brown D, Kovacheva EL, Gonzalez-Cadavid NF, Sinha-Hikim I. Involvement of oxidative stress and caspase 2-mediated intrinsic pathway signaling in age-related increase in muscle cell apoptosis in mice. Apoptosis. 2008;13:822–832. doi: 10.1007/s10495-008-0216-7 18461459 PMC4732709

[36] Rom O, Kaisari S, Aizenbud D, Reznick AZ. The effects of acetaldehyde and acrolein on muscle catabolism in C2 myotubes. Free Radic Biol Med. 2013;65:190–200. doi: 10.1016/j.freeradbiomed.2013.06.024 23792774

[37] Zelko IN, Dassanayaka S, Malovichko MV, Howard CM, Garrett LF, Uchida S, Brittian KR, Conklin DJ, Jones SP, Srivastava S. Chronic Benzene Exposure Aggravates Pressure Overload-Induced Cardiac Dysfunction. Toxicol Sci. 2021;185:64–76. doi: 10.1093/toxsci/kfab125 34718823 PMC8714365

[38] Watson LJ, Facundo HT, Ngoh GA, Ameen M, Brainard RE, Lemma KM, Long BW, Prabhu SD, Xuan YT, Jones SP. O-linked beta-N-acetylglucosamine transferase is indispensable in the failing heart. Proc Natl Acad Sci U S A. 2010;107:17797–17802. doi: 10.1073/pnas.1001907107 20876116 PMC2955091

[39] Hoetker D, Chung W, Zhang D, Zhao J, Schmidtke VK, Riggs DW, Derave W, Bhatnagar A, Bishop DJ, Baba SP. Exercise alters and beta-alanine combined with exercise augments histidyl dipeptide levels and scavenges lipid peroxidation products in human skeletal muscle. J Appl Physiol (1985). 2018. doi: 10.1152/japplphysiol.00007.2018 PMC10392632 30335580

[40] Hajahmadi M, Shemshadi S, Khalilipur E, Amin A, Taghavi S, Maleki M, Malek H, Naderi N. Muscle wasting in young patients with dilated cardiomyopathy. J Cachexia Sarcopenia Muscle. 2017;8:542–548. doi: 10.1002/jcsm.12193 28251827 PMC5566643

[41] von Haehling S, Steinbeck L, Doehner W, Springer J, Anker SD. Muscle wasting in heart failure: An overview. Int J Biochem Cell Biol. 2013;45:2257–2265. doi: 10.1016/j.biocel.2013.04.025 23665153

[42] Zamboni M, Rossi AP, Corzato F, Bambace C, Mazzali G, Fantin F. Sarcopenia, cachexia and congestive heart failure in elderly individuals. Endocr Metab Immune Disord Drug Targets. 2013;13:58–67. doi: 10.2174/1871530311313010008 23369138

[43] Szaroszyk M, Kattih B, Martin-Garrido A, Trogisch FA, Dittrich GM, Grund A, Abouissa A, Derlin K, Meier M, Holler T, Skeletal muscle derived Musclin protects the heart during pathological overload. Nat Commun. 2022;13:149. doi: 10.1038/s41467-021-27634-5 35013221 PMC8748430

[44] Anker SD, Steinborn W, Strassburg S. Cardiac cachexia. Ann Med. 2004;36:518–529. doi: 10.1080/07853890410017467 15513302

[45] Adigun AQ, Ajayi AA. The effects of enalapril-digoxin-diuretic combination therapy on nutritional and anthropometric indices in chronic congestive heart failure: preliminary findings in cardiac cachexia. Eur J Heart Fail. 2001;3:359–363. doi: 10.1016/s1388-9842(00)00146-x 11378008

[46] Chamberlain JS. ACE inhibitor bulks up muscle. Nat Med. 2007;13:125–126. doi: 10.1038/nm0207-125 17290265

[47] Moulin M, Ferreiro A. Muscle redox disturbances and oxidative stress as pathomechanisms and therapeutic targets in early-onset myopathies. Semin Cell Dev Biol. 2017;64:213–223. doi: 10.1016/j.semcdb.2016.08.003 27531051

[48] Chen HJ, Wang CC, Chan DC, Chiu CY, Yang RS, Liu SH. Adverse effects of acrolein, a ubiquitous environmental toxicant, on muscle regeneration and mass. J Cachexia Sarcopenia Muscle. 2019;10:165–176. doi: 10.1002/jcsm.12362 30378754 PMC6438343

[49] Nakashima Y, Ohsawa I, Nishimaki K, Kumamoto S, Maruyama I, Suzuki Y, Ohta S. Preventive effects of Chlorella on skeletal muscle atrophy in muscle-specific mitochondrial aldehyde dehydrogenase 2 activity-deficient mice. BMC Complement Altern Med. 2014;14:390. doi: 10.1186/1472-6882-14-390 25305781 PMC4200191

[50] Moghaddam AE, Gartlan KH, Kong L, Sattentau QJ. Reactive carbonyls are a major Th2-inducing damage-associated molecular pattern generated by oxidative stress. J Immunol. 2011;187:1626–1633. doi: 10.4049/jimmunol.1003906 21742965

[51] Di Gioia M, Spreafico R, Springstead JR, Mendelson MM, Joehanes R, Levy D, Zanoni I. Endogenous oxidized phospholipids reprogram cellular metabolism and boost hyperinflammation. Nat Immunol. 2020;21:42–53. doi: 10.1038/s41590-019-0539-2 31768073 PMC6923570

[52] Ngwenyama N, Kirabo A, Aronovitz M, Velazquez F, Carrillo-Salinas F, Salvador AM, Nevers T, Amarnath V, Tai A, Blanton RM, Isolevuglandin-Modified Cardiac Proteins Drive CD4+ T-Cell Activation in the Heart and Promote Cardiac Dysfunction. Circulation. 2021;143:1242–1255. doi: 10.1161/CIRCULATIONAHA.120.051889 33463362 PMC7987774

[53] Miller YI, Choi SH, Wiesner P, Fang L, Harkewicz R, Hartvigsen K, Boullier A, Gonen A, Diehl CJ, Que X, Oxidation-specific epitopes are danger-associated molecular patterns recognized by pattern recognition receptors of innate immunity. Circ Res. 2011;108:235–248. doi: 10.1161/CIRCRESAHA.110.223875 21252151 PMC3075542

[54] Kobayashi H, Nakamura S, Sato Y, Kobayashi T, Miyamoto K, Oya A, Matsumoto M, Nakamura M, Kanaji A, Miyamoto T. ALDH2 mutation promotes skeletal muscle atrophy in mice via accumulation of oxidative stress. Bone. 2021;142:115739. doi: 10.1016/j.bone.2020.115739 33188956

[55] Kasai A, Jee E, Tamura Y, Kouzaki K, Kotani T, Nakazato K. Aldehyde dehydrogenase 2 deficiency promotes skeletal muscle atrophy in aged mice. Am J Physiol Regul Integr Comp Physiol. 2022;322:R511–R525. doi: 10.1152/ajpregu.00304.2021 35318866

[56] Zhang Q, Zheng J, Qiu J, Wu X, Xu Y, Shen W, Sun M. ALDH2 restores exhaustive exercise-induced mitochondrial dysfunction in skeletal muscle. Biochem Biophys Res Commun. 2017;485:753–760. doi: 10.1016/j.bbrc.2017.02.124 28249782

[57] Fu SH, Zhang HF, Yang ZB, Li TB, Liu B, Lou Z, Ma QL, Luo XJ, Peng J. Alda-1 reduces cerebral ischemia/reperfusion injury in rat through clearance of reactive aldehydes. Naunyn Schmiedebergs Arch Pharmacol. 2014;387:87–94. doi: 10.1007/s00210-013-0922-8 24081521

[58] Woods C, Shang C, Taghavi F, Downey P, Zalewski A, Rubio GR, Liu J, Homburger JR, Grunwald Z, Qi W, In Vivo Post-Cardiac Arrest Myocardial Dysfunction Is Supported by Ca2+/Calmodulin-Dependent Protein Kinase II-Mediated Calcium Long-Term Potentiation and Mitigated by Alda-1, an Agonist of Aldehyde Dehydrogenase Type 2. Circulation. 2016;134:961–977. doi: 10.1161/CIRCULATIONAHA.116.021618 27582424 PMC5040468

[59] Gomes KM, Campos JC, Bechara LR, Queliconi B, Lima VM, Disatnik MH, Magno P, Chen CH, Brum PC, Kowaltowski AJ, Aldehyde dehydrogenase 2 activation in heart failure restores mitochondrial function and improves ventricular function and remodeling. Cardiovasc Res. 2014;103:498–508. doi: 10.1093/cvr/cvu125 24817685 PMC4155470

[60] Drozak J, Chrobok L, Poleszak O, Jagielski AK, Derlacz R. Molecular identification of carnosine N-methyltransferase as chicken histamine N-methyltransferase-like protein (hnmt-like). PLoS One. 2013;8:e64805. doi: 10.1371/journal.pone.0064805 23705015 PMC3660329

[61] Drozak J, Piecuch M, Poleszak O, Kozlowski P, Chrobok L, Baelde HJ, de Heer E. UPF0586 Protein C9orf41 Homolog Is Anserine-producing Methyltransferase. J Biol Chem. 2015;290:17190–17205. doi: 10.1074/jbc.M115.640037 26001783 PMC4498059

[62] Drozak J, Veiga-da-Cunha M, Vertommen D, Stroobant V, Van Schaftingen E. Molecular identification of carnosine synthase as ATP-grasp domain-containing protein 1 (ATPGD1). J Biol Chem. 2010;285:9346–9356. doi: 10.1074/jbc.M109.095505 20097752 PMC2843183

[63] Boldyrev AA, Aldini G, Derave W. Physiology and pathophysiology of carnosine. Physiol Rev. 2013;93:1803–1845. doi: 10.1152/physrev.00039.2012 24137022

[64] Aldini G, Granata P, Carini M. Detoxification of cytotoxic alpha, beta-unsaturated aldehydes by carnosine: characterization of conjugated adducts by electrospray ionization tandem mass spectrometry and detection by liquid chromatography/mass spectrometry in rat skeletal muscle. J Mass Spectrom. 2002;37:1219–1228. doi: 10.1002/jms.381 12489081

[65] Baguet A, Koppo K, Pottier A, Derave W. Beta-alanine supplementation reduces acidosis but not oxygen uptake response during high-intensity cycling exercise. Eur J Appl Physiol. 2010;108:495–503. doi: 10.1007/s00421-009-1225-0 19841932

[66] Posa DK, Miller J, Hoetker D, Ramage MI, Gao H, Zhao J, Doelling B, Bhatnagar A, Wigmore SJ, Skipworth RJE, Baba SP. Skeletal muscle analysis of cancer patients reveals a potential role for carnosine in muscle wasting. J Cachexia Sarcopenia Muscle. 2023. doi: 10.1002/jcsm.13258 PMC10401540 37199284

[67] de Courten B, Jakubova M, de Courten MP, Kukurova IJ, Vallova S, Krumpolec P, Valkovic L, Kurdiova T, Garzon D, Barbaresi S, Effects of carnosine supplementation on glucose metabolism: Pilot clinical trial. Obesity (Silver Spring). 2016;24:1027–1034. doi: 10.1002/oby.21434 27040154

[68] Lombardi C, Carubelli V, Lazzarini V, Vizzardi E, Bordonali T, Ciccarese C, Castrini AI, Dei Cas A, Nodari S, Metra M. Effects of oral administration of orodispersible levo-carnosine on quality of life and exercise performance in patients with chronic heart failure. Nutrition. 2015;31:72–78. doi: 10.1016/j.nut.2014.04.021 25287762

[69] Everaert I, De Naeyer H, Taes Y, Derave W. Gene expression of carnosine-related enzymes and transporters in skeletal muscle. Eur J Appl Physiol. 2013;113:1169–1179. doi: 10.1007/s00421-012-2540-4 23124893

